# Gestational Zearalenone Exposure Causes Reproductive and Developmental Toxicity in Pregnant Rats and Female Offspring

**DOI:** 10.3390/toxins9010021

**Published:** 2017-01-05

**Authors:** Xin Gao, Lvhui Sun, Niya Zhang, Chong Li, Jiacai Zhang, Zhuohui Xiao, Desheng Qi

**Affiliations:** 1 College of Animal Nutrition and Feed Science, Huazhong Agricultural University, Wuhan 430070, Hubei, China; gaoxinnutrition@webmail.hzau.edu.cn (X.G.); lvhuisun@mail.hzau.edu.cn (L.S.); zhangniya@mail.hzau.edu.cn (N.Z.); chrisli@webmail.hzau.edu.cn (C.L.); zjcc404@126.com (J.Z.); crystal_cuke@163.com (Z.X.); 2 The Cooperative Innovation Center for Sustainable Pig Production, Wuhan 430070, Hubei, China

**Keywords:** zearalenone, gestational exposure, transgenerational toxicity, F1 female rats

## Abstract

Zearalenone (ZEN) is an oestrogenic mycotoxin commonly found in food and feed products and can affect reproduction and development in both humans and animals. This study aimed to determine the toxic effects of ZEN on maternal SD rats and the F1 female offspring. Sixty-four pregnant rats were divided into 4 groups and exposed to feed contaminated with ZEN (0, 5, 10, and 20 mg/kg feed) on gestational days (GDs) 0–21. Compared with the controls, the groups exposed to 10 and 20 mg/kg ZEN showed significantly decreased feed intake and body weight of pregnant rats and/or female offspring. Meanwhile, 20 mg/kg ZEN significantly decreased the birth weight and viability of F1 newborn rats. Moreover, 10 and 20 mg/kg ZEN diets increased follicle-stimulating hormone concentrations but decreased oestradiol in both maternal and F1 adult rats. In the F1 generation, ZEN caused no pathological changes in ovaries and uterus in weaned rats, but significant follicular atresia and a thinning uterine layer were found in F1 female adult rats in the 20 mg/kg ZEN group. These impairments concurred with the inhibited mRNA and protein levels of oestrogen receptor-alpha (Esr1) and 3β-hydroxysteroid dehydrogenase (HSD) in the adult uterus and/or ovaries. Furthermore, 10 and/or 20 mg/kg ZEN exposure significantly reduced Esr1, gonadotropin-releasing hormone receptor (GnRHr), and ATP binding cassette transporters b1 and c1 (ABCb1 and ABCc1) in the placenta and foetal and weaned F1 brains, and also produced a dose-dependent increase in 3β-HSD in the placenta. Additionally, 20 mg/kg ZEN significantly upregulated ABCc5 expression in the placenta and ovaries of weaned rats. These results suggested that prenatal ZEN exposure in rats affected maternal and foetal development and may lead to long-term reproductive impairment in F1 adult females.

## 1. Introduction

Zearalenone (ZEN), also known as F-2 toxin, is a secondary metabolite produced by *Fusarium* species, and is a nonsteroidal oestrogenic mycotoxin that is commonly found in cereals worldwide [1,2,3]. Animal consumption of feed contaminated with ZEN can cause serious defects in health, growth, and reproductive performance. Swine are the most sensitive and most frequently affected animals [3,4]. After intake, ZEN is predominantly metabolized to α- and β-zearalenol (ZOL) by 3α- and 3β-hydroxysteroid dehydrogenases (HSDs) in the liver, and α-ZOL has been shown to be the dominant ZEN derivative in pigs, humans, rats, and mice [5,6]. Due to the structural similarity with 17β-oestradiol (E_2_), ZEN can bind to the oestrogen receptor, and the receptor–ZEN conjugate translocates into the nucleus, where it binds to oestrogen response elements (EREs), activating target gene transcription and generating an oestrogen-like response in many organs [7,8,9,10]. In humans, prepubertal consumption of ZEN has been related with occurrence of precocious puberty in girls [11]. Females are more sensitive to ZEN than males, and several in vivo studies have reported the toxicological effects of ZEN—including functional alterations in reproductive organs, increased embryo-lethal resorption, decreased fertility, and abnormal hormone levels—on the female reproductive system in laboratory and farm animals [12,13,14].

ZEN has been associated with adverse effects on reproductive function in different species; however, the adverse effects may be even more pronounced during gestation, as the foetus is highly susceptible to toxins due to its incomplete immune system [11,15]. Exposure of the mother to environmental toxic substances during pregnancy can cause in utero foetal stress and ultimately contribute to long-term growth and developmental problems throughout the lifetime of the neonate [16,17]. Previous studies have indicated that exposure to endocrine disruptors, such as bisphenol A and dioxin, during gestation could causes abnormal development of the reproductive system in female animals [18]. Likewise, ZEN and its metabolites are transferred to the foetus by the placenta, and associations have been identified between maternal ZEN exposure and adverse pregnancy outcomes, such as impaired development and reduced litter size. Moreover, foetal malformation can be caused by excessive ZEN administration to the mother [19,20,21]. After maternal xenobiotic exposure, reports have shown a comparatively higher susceptibility in female neonates than that of male [22,23]. Foetal exposure to ZEN may cause follicle damage and therefore comprises a health risk for young females due to premature oocyte depletion [23]. Because of the importance of ZEN as a major mycotoxin worldwide, it is necessary to determine the transgenerational reproductive and developmental toxicity in animals, especially domestic animals.

Despite extensive reports on the adverse effects of ZEN on reproductive toxicity in various animals, the transgenerational toxicity of ZEN has not been fully elucidated. Previous studies have focused on one time point and reported different results, while limited information is known about the effects of ZEN on development and reproduction of both maternal animals and F1 offspring. Therefore, the present study used rats as an animal model and attempted to investigate (1) the reproductive toxicity of ZEN in pregnant rats after gestational exposure and (2) whether prenatal exposure to ZEN was associated with impaired reproduction and development in adult female offspring.

## 2. Results

### 2.1. ZEN Effects on Development and Reproduction of Pregnant Rats

The effects of dietary ZEN on feed consumption, body weight (BW) gain, and reproductive performance of pregnant rats are shown in Table 1. The BW of the animals in 4 groups was similar at the beginning of the experiment, while on gestational day (GD) 21, the BW decreased (*p* < 0.05) in the 10 and 20 ZEN groups. During gestation, the average daily intake (ADI) decreased significantly in the 20 ZEN group, and average daily gain (ADG) decreased (*p* < 0.05) in both the 10 and 20 ZEN groups. Pregnant rats treated with ZEN at concentrations up to 20 mg/kg showed no significant effect on litter size. However, 20 mg/kg ZEN significantly decreased the number of liveborn pups (*p* < 0.05) compared to that of the controls. Meanwhile, the viability of newborn pups decreased (*p* < 0.05) in a ZEN dose-dependent manner.

### 2.2. Effects on BW and Growth Performance of F1 Female Rats

Decreases (*p* < 0.05) in BW were observed in neonatal and weaned F1 female rats prenatally treated with 20 mg/kg ZEN compared with that of the control (Table 2). For adult F1 females, ZEN significantly decreased BW (*p* < 0.05) in a dose-dependent manner. The ADI and ADG of F1 female rats from postnatal day (PND) 21–63 are shown in Table 2. While no significant effects on ADI were found, a diet of ZEN up to 20 mg/kg decreased (*p* < 0.05) the ADG of F1 female rats during PND 21–63.

### 2.3. Effects on Serum Hormone Levels of Maternal and F1 Adult Rats

The concentrations of follicle-stimulating hormone (FSH), luteinizing hormone (LH), and sex steroid oestradiol (E_2_) in the serum of maternal and F1 adult female rats on PND 63 were analysed as shown in Table 3. The results showed a statistically significant decrease (*p* < 0.05) in FSH for maternal rats on GD 20; meanwhile, a significant increase (*p* < 0.05) in E_2_ concentration in the 20 ZEN group was noted. For adult female rats, both LH and FSH showed increases (*p* < 0.05) in the 20 ZEN group compared to those of the control, while the E_2_ levels decreased (*p* < 0.05) in a dose-dependent manner in the ZEN 5 through ZEN 20 groups.

### 2.4. Histopathological Analysis of F1 Reproductive Organs

**Figure 1:**
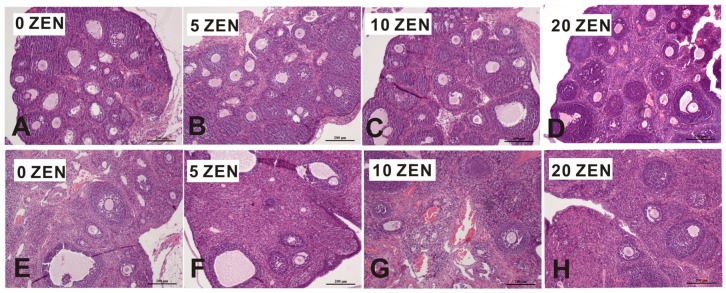
Effects of prenatal ZEN exposure on ovaries of F1 weaned and adult female rats. The hepatic sections were stained with haematoxylin and eosin; photomicrographs are shown at 100 or 200× magnification. Weaned ovaries: (**A**–**D**) adult ovaries: (**E**–**H**) ZEN, zearalenone; Control, 0 ZEN diet; 5 ZEN, 5 mg/kg ZEN diet; 10 ZEN, 10 mg/kg ZEN diet; 20 ZEN, 20 mg/kg ZEN diet.

The histopathological analysis revealed pathological changes in the ovaries and uterus of weaned and adult F1 rats. Figure 1 shows numerous ovarian follicles at different stages of development in F1 weaned and adult rats. No obvious differences were observed in ovaries from the weaned rats, and many ovarian follicles at different stages were found in all groups (Figure 1A–D). For adult female rats, control, 5 ZEN, and 10 ZEN treatments had normal ovarian follicles (Figure 1E–G); however, in the 20 ZEN treatment, significant increases in abnormal ovarian follicles and nonfunctional follicles were observed (Figure 1H).

**Figure 2:**
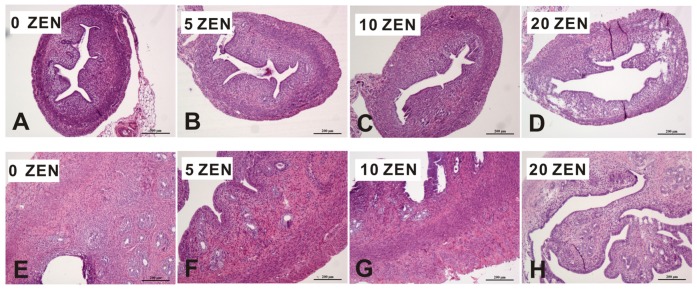
Effects of prenatal ZEN exposure on uterus of F1 weaned and adult female rats. The hepatic sections were stained with haematoxylin and eosin; photomicrographs are shown at 100 or 200× magnification. Weaned uterus: (**A**–**D**) adult uterus: (**E**–**H**) ZEN, zearalenone; Control, 0 ZEN diet; 5 ZEN, 5 mg/kg ZEN diet; 10 ZEN, 10 mg/kg ZEN diet; 20 ZEN, 20 mg/kg ZEN diet.

In the uterus of weaned rats, no structural differences were observed in the control, 5 ZEN, and 10 ZEN groups (Figure 2A–C), while thinning of the uterine layer was noted in the 20 ZEN group (Figure 2D). In the uterus of adult rats, there were many actively proliferating cells around the uterine glands in the control (Figure 2E), which was also observed in the 5 ZEN group (Figure 2F); in the 10 ZEN group, there were fewer submucosal glands compared to those of control group, and glandular epithelial cell death and infiltrating eosinophils were also observed (Figure 2G); 20 ZEN samples exhibited significant mucosal hyperplasia and muscular layer thinning compared to those of the control group (Figure 2H).

### 2.5. Effects on mRNA Expressions of ABC Transporters and Hormone Related Genes

**Figure 3:**
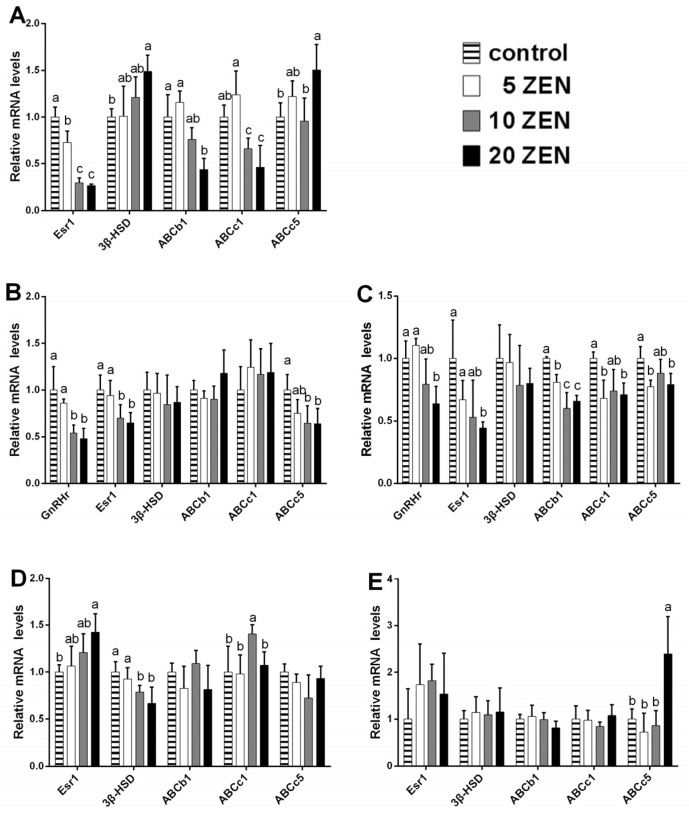
Effects of prenatal ZEN exposure on relative mRNA abundance of hormones-related genes or ABC transporters in placenta, foetal brain, and weaned F1 tissues. Values are means ± SD, *n* = 12. ^a,b,c^ Means without a common letter differ, *p* < 0.05. ZEN, zearalenone; Esr1, oestrogen receptor alpha; GnRHr, gonadotropin-releasing hormone receptor; 3β-HSD, 3β-hydroxysteroid dehydrogenase; ABCb1, ATP binding cassette transporters b1; ABCc1, ATP binding cassette transporters c1; ABCc5, ATP binding cassette transporters c5. (**A**) Placenta; (**B**) foetal brain; (**C**) weaned F1 brain; (**D**) weaned F1 uterus; (**E**) weaned F1 ovary.

**Figure 4:**
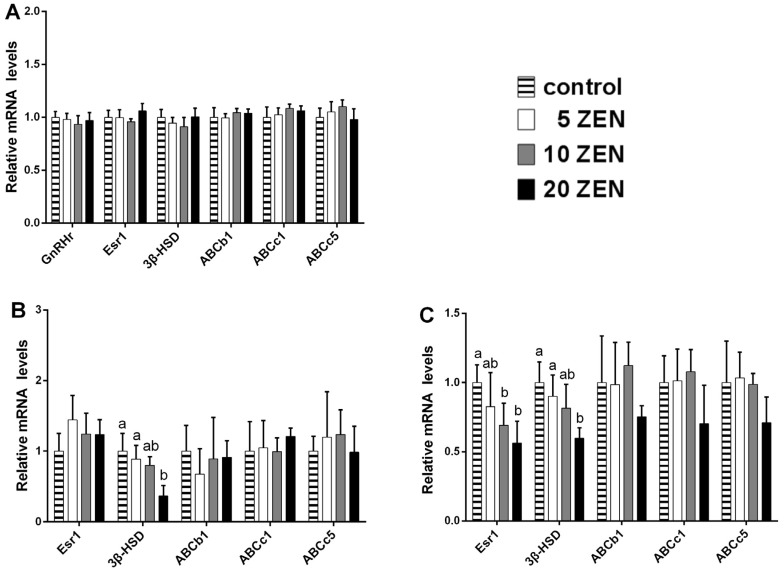
Effects of prenatal ZEN exposure on relative mRNA abundance of hormones-related genes or ABC transporters in brain, uterus, and ovary in adult female F1 rats. Values are means ± SD, *n* = 12. ^a,b,c^ Means without a common letter differ, *p* < 0.05. ZEN, zearalenone; Esr1, oestrogen receptor alpha; GnRHr, gonadotropin-releasing hormone receptor; 3β-HSD, 3β-hydroxysteroid dehydrogenase; ABCb1, ATP binding cassette transporters b1; ABCc1, ATP binding cassette transporters c1; ABCc5, ATP binding cassette transporters c5. (**A**) Adult F1 brain; (**B**) adult F1 uterus; (**C**) adult F1 ovary.

In the placenta, significant 3-fold inhibitions (*p* < 0.05) of the mRNA expression of oestrogen receptor-alpha (Esr1) were observed in both the 10 ZEN and 20 ZEN treatments. Similarly, downregulation (*p* < 0.05) of the ATP binding cassette transporters b1 and c1 (ABCb1 and ABCc1) was found in the 20 ZEN treatments (more than 2-fold). Additionally, a significant 1.5-fold increase (*p* < 0.05) in ABCc5 was noted in the 20 ZEN group (Figure 3A). In foetal brains, downregulation (*p* < 0.05) of the gonadotropin-releasing hormone receptor (GnRHr), Esr1, and ABCc5 was observed in both the 10 ZEN and 20 ZEN treatments. In the brains of weaned female rats, GnRHr, Esr1, ABCb1, ABCc1, and ABCc5 were significantly inhibited (*p* < 0.05) in the 20 ZEN group (Figure 3C). In the uterus of weaned rats, Esr1 was upregulated (*p* < 0.05) in the 20 ZEN treatment group (1.4-fold increase); however, significant downregulation (*p* < 0.05) of 3β-HSD mRNA was observed in both the 10 ZEN and 20 ZEN groups (1.4- and 1.7-fold, respectively). Additionally, a slight induction of ABCc1 in the 10 ZEN group was found in the uterus of weaned rats (Figure 3D). In the ovaries of weaned rats, a 2.5-fold increase (*p* < 0.05) in ABCc5 was observed in the 20 ZEN group (Figure 3E). For adult rats, no significant effect was found in all genes detected in the brain (Figure 4A). In the uterus, downregulation (*p* < 0.05) of 3β-HSD mRNA was noted in the 20 ZEN groups (more than 2.5-fold), but no significant effects were found for Esr1 and ABC transporters (Figure 4B). In adult ovaries, significant inhibition (*p* < 0.05) of Esr1 and 3β-HSD mRNA (almost 2-fold for both genes) was noted in the 20 ZEN group (Figure 4C).

### 2.6. Effects on Protein Expressions of 3β-HSD and Esr1 in Adult Ovaries and Uterus

**Figure 5:**
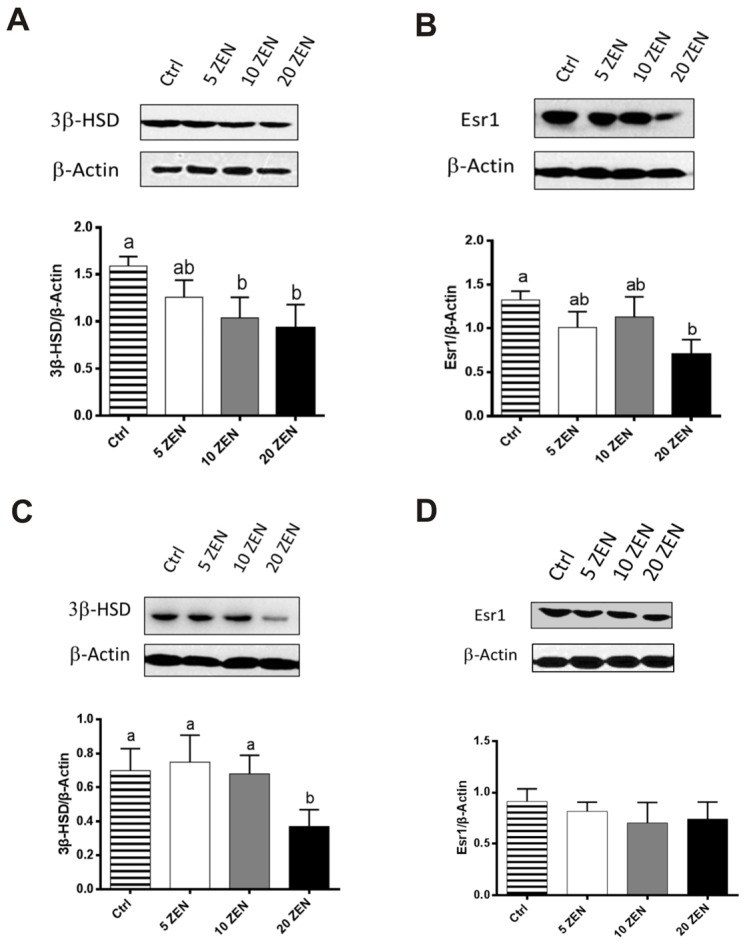
Effects of prenatal ZEN exposure on protein expressions of 3β-HSD and Esr1 in ovaries and uterus in adult female F1 rats. Values are means ± SD, *n* = 6. ^a,b,c^ Means without a common letter differ, *p* < 0.05. ZEN, zearalenone; Esr1, oestrogen receptor alpha; 3β-HSD, 3β-hydroxysteroid dehydrogenase. (**A**) 3β-HSD expressions in adult F1 ovaries; (**B**) Esr1 expressions in adult F1 ovaries; (**C**) 3β-HSD expressions in adult F1 uterus; (**D**) Esr1 expressions in adult F1 uterus.

Western blotting experiments of 3β-HSD and Esr1 were performed on the ovaries and uterus in adult F1 female rats, since they were most susceptible tissues. The modulation of protein expressions followed mRNA modulation in the ovaries and uterus (Figure 5). Protein expression of 3β-HSD was 1.5- and 1.7-fold inhibited (*p* < 0.05) in adult ovaries in 10 ZEN and 20 ZEN groups, respectively (Figure 5A). Also, in adult uterus, a 1.9-fold inhibition was found for 3β-HSD protein (Figure 5B). For Esr1 protein, a significant 1.9-fold inhibition was observed in adult ovaries, but no significant effect was noted for Esr1 in adult uterus (Figure 5D).

## 3. Discussion

The present study revealed that the effects of maternal oral ZEN exposure during pregnancy were not limited to the duration of gestation, but also caused long-term transgenerational toxicity on development and reproduction of female offspring. In the present study, rats exposed to ZEN through the diet at 10 and/or 20 mg/kg during gestation showed significant decreases not only in maternal BW, ADI, and ADG but also in the BW and ADG of the female offspring. Consumption of ZEN did not affect litter size in the present study, while the survival rate of newborn rats significantly decreased in the 20 ZEN group. These results are consistent with previous reports demonstrating that early gestational ZEN exposure influenced maternal development and foetus mortality in rats [20,24]. In the present experiments, 5 mg/kg ZEN caused no significant reproductive effects in both maternal and F1 female rats, which is consistent with the reported ZEN no-observed effect level (NOEL) in rats [12]. Low birth weight is a serious problem in livestock production, and it has been associated with neonatal mortality and morbidity, as well as increased risk for the development of diseases later in life [25]. In the present study, a significant decrease in birth weight was found in newborn rats in the 20 ZEN group compared to that of the control group, which is consistent with previous studies in rats and pigs [26,27,28]. The delayed development of offspring in the ZEN-treated groups may be associated with the low birth weight. These data indicate that ZEN gestational exposure could cause transgenerational toxicity through dams to offspring, resulting in developmental dysfunction in young females.

Gestation is a hormone-sensitive period for both the mother and the foetus, and altering hormone levels by dietary xenobiotics is a concern. Similar to the previous reports in rats [12,20], in maternal rats, we observed significant increases in FSH and decreases in E_2_ on GD 20 after ZEN exposure, while no significant changes in LH were observed. For adult F1 female rats, 20 mg/kg ZEN significantly increased FSH and LH, while it decreased E_2_ levels. These sexual hormone changes in F1 rats are considered a sign of premature ovarian failure [29,30]. The reduction in E_2_ may be due to the structural changes in the ovaries with increased atresia of ovarian follicle granulosa cells [31], which was also observed in the present study. Meanwhile, a thinning uterine layer with reduced uterus weight (Supplementary Table S1) was also found in the 20 ZEN offspring, and these changes may be associated with decreased E_2_ secretion and impaired uterine tissue morphology [7]. However, no significant pathological changes were observed in weaned rats on PND 21 except a thinning uterine wall in the 20 ZEN group. This may be because the intensity of intoxication symptoms is determined by the degree of the animal’s sexual maturity and by the functioning of its reproductive system, which have been reported in pigs and rams [23,32,33]. These effects induced by ZEN indicate a risk of sexual hormone dysfunction and reproductive dysplasia in the F1 female generation. In livestock production, these effects may seriously affect the breeding value of the F1 generation, and further studies should be performed to assess the reproductive function of prenatal ZEN-exposed offspring.

ABC transporters are expressed in various tissues and influence oral bioavailability and efflux clearance of multiple xenobiotics [34]. In the present study, the transgenerational toxicity induced by the ZEN diet in the dams and offspring was associated with downregulation of ABCb1 and ABCc1 mRNA in the placenta and foetal and weaned F1 brains, which is consistent with previous reports in rats [35,36]. ABCb1 (also known as P-glycoprotein) and ABCc1 have been shown to play important roles in the utero–placental transport signalling [37,38]. Early studies have indicated that ABCb1, ABCc1, and ABCc5 are directly involved in protecting tissues from xenobiotic accumulation and the resulting toxicity; decreases in their expression may have deleterious consequences on foetal development [39,40]. Moreover, studies have shown that exposure to Cd, Hg, Pb, and as significantly induced the mRNA expression of ABCc5 in ZF4 cells and zebrafish embryos [41]. Similarly, ABCc5 expression was significantly induced in 20 ZEN placenta and weaned F1 ovaries. In the present study, brains of foetal and weaned female rats showed greater downregulation of ABC transporters than that of the other analysed tissues. Interestingly, no statistically significant changes were observed in the brains of adult females. Our results indicated that ZEN may transport through the blood–brain barrier and cause negative effects on early brain development; thus, the intact blood–brain barrier and abilities of ABC transporters to modulate the absorption and elimination of ZEN may reduce these toxic effects on the brain [42,43,44].

Prenatal ZEN exposure also affected hormone-related genes and proteins expression in various tissues of F1 female rats. Interestingly, in contrast to previous studies that reported increased Esr1 expression after direct ZEN exposure [20,26], the present study showed decreased Esr1 mRNA expression in the placenta and brain of foetal or weaned female rats, as well as consistent decreased Esr1 mRNA and protein expressions in mature ovaries. These may indicate a feedback mechanism that includes transcriptional and translational repression of Esr1 [45]. Esr1 is a known ZEN target, and cross-talk is observed in the signalling pathways of Esrs [9,46]. Therefore, the decrease in Esr1 may indicate a decreased ability to mediate the biological effect of oestrogens and result to reproductive impairments [47,48]. Our results also showed significant decrease of GnRHr mRNA expression in foetal and weaned brains in the 20 ZEN, indicating disturbed GnRH activation. Impaired GnRH activation and dysfunction hormone secretion may lead to disruption of female reproductive physiology [26,49]. Previous studies have reported that ZEN is a substrate for 3β-HSD, which is highly expressed in the placenta, foetus, and brain of various animal species [50,51]. The significant increase in 3β-HSD expression in foetal brains in the present study may indicate the brain toxicity of ZEN, or it may be an adaptive response by the brain to protect itself from acute infection-mediated inflammation [52]. The present study also showed a notable decrease of 3β-HSD in adult F1 uterus and ovaries in both mRNA and protein levels, and this may explain the decrease of E_2_ levels in F1 adult females. Reduction of 3β-HSD, an important steroidogenesis pathway enzyme, may affect the synthesis of E_2_, leading to abnormal changes in gonadal function [53,54].

## 4. Conclusions

In summary, the present experiments in prenatal ZEN-exposed rats demonstrate that this toxin, which is capable of inducing maternal and F1 growth restriction and hormonal dysfunction, also induces structural abnormalities in the uterus and ovaries of the adult offspring. In addition, our results showed that at least six genes (ABCb1, ABCc1, ABCc5, Esr1, GnRHr, and 3β-HSD) and two proteins (Esr1 and 3β-HSD) were involved in these transgenerational toxicities. The negative effects on female offspring include decreased reproductive functions, which may affect conception and procreation. For humans, pregnant exposure to ZEN-contaminated food might therefore comprise a health risk for both mother and the young female generation due to the transgenerational toxicity. Although this report provides preliminary evidence for an association between ZEN and offspring development, further studies are needed to elucidate the precise signalling mechanisms through which ZEN affects gonadal function in F1 offspring.

## 5. Materials and Methods

### 5.1. Animals and Experimental Procedures

All animal experimental procedures were performed in accordance with the National Research Council Guide [55], and approved by the Scientific Ethic Committee of Huazhong Agricultural University on 7 August 2015. The project identification code is HZAURA-2015-006.

ZEN was purchased from Sigma-Aldrich (St. Louis, MO, USA). Sprague-Dawley (SD) rats were obtained from the Wuhan Centers for Disease Prevention & Control (Wuhan, China) and were acclimated for 1 week prior to experimentation. The rats weighted approximately 210 g and 300 g for females and males, respectively. The male rats were not treated and used as sires only. After mating, pregnant SD rats at GD 0 were individually housed in temperature-controlled rooms with 12 h light/dark and given free access to water and feed. A total of 64 pregnant rats were divided into 4 groups with 16 replicates of 1 rat per cage and received diets containing different concentrations (0.01, 4.82, 9.21, 19.54 mg/kg) of ZEN from GD 0–20. The 4 groups were named as follows: *Control*, *5 ZEN*, *10 ZEN*, and *20 ZEN* (Supplementary Table S2). The ZEN concentrations were based on previous studies [3,56]. The pregnant rats were fed diets containing ZEN throughout the gestational period, but not during the lactation phase of the study. Feed intake and BW gain of pregnant rats were recorded weekly during gestation.

Eight pregnant rats in each group were sacrificed on GD 21 by cervical dislocation, and foetal brains were collected (*n* = 12). The remaining 8 pregnant rats in each group were allowed to deliver and nurse normally. At birth, the litter size and viability of foetuses were recorded. Each pup was sexed, weighed, and identified. The litter size was balanced to 8 with half males and females. All pups were nursed and weaned on PND 21. Only the F1 female rats were investigated in the present study. F1 female rats were sacrificed by cervical dislocation on PND 20 (*n* = 12, two pups each litter). The remaining female offspring were divided into 4 groups of 3 rats per cage. These F1 female rats were fed a normal diet until they matured and were then sacrificed (*n* = 12) on PND 63 (9 weeks), which was considered sexually mature. The time points selected in the present study were based on previous reports [57,58]. Blood samples of adult F1 female rats, as well as the uterus and ovaries of weaned and adult rats, were collected for hormone and histological examinations as previously described [59].

### 5.2. Hormone Levels Detection

FSH, LH, and E_2_ of maternal and F1 adult rats were determined using enzyme-linked immunosorbent assay kits (R&D Systems, Inc., Minneapolis, MO, USA) according to the manufacturer’s recommended protocols.

### 5.3. Histopathological Analyses

The ovaries and uterus of weaned and adult F1 rats were fixed in 10% neutral-buffered formalin. The centre of each organ was processed for paraffin embedding, sectioned (thickness, 5 mm), and stained with haematoxylin and eosin (H&E) [15]. The slides were observed under 100 or 200× magnification using an optical microscope (Nikon, Tokyo, Japan).

### 5.4. Total RNA Extraction and qPCR 

Total mRNA was extracted from the organs with TRIzol (Invitrogen, Carlsbad, CA, USA) according to the manufacturer’s protocol. The RNA quality and concentration were estimated by a nucleic acid concentration analyser (NanoDrop 2000, Thermo Fisher, Waltham, MA, USA). Expression levels of six genes (Esr1, GnRHr, 3β-HSD, and ATP binding cassette transporters (ABCb1, ABCc1 and ABCc5)) were analysed using real-time qPCR (CFX384, Bio-Rad, Hercules, CA, USA). The 2^−ddCt^ method was used for quantification with glyceraldehyde 3-phosphate dehydrogenase (GADPH) as a reference gene, as previously described [60]. Primers (Supplementary Table S3) used in the study were designed with Primer Express 3.0 (Applied Biosystems, Foster, CA, USA).

### 5.5. Western Blotting Analyses

Proteins were extracted from the uterus and ovaries of adult F1 female rats with a Tissue Total Protein Extraction Kit (Sangon Biotech, Shanghai, China). Western blot was performed as described by Farah et al. [36]. The primary antibodies used in the present study were: Esr1 (Abcam ab32063/1:1000, Cambridge, MA, USA), 3β-HSD (Santa Cruz sc-30820/1:500, Santa Cruz, CA, USA), and β-actin (Cell Signaling Technology #4967/1:2000, Boston, MA, USA). Secondary horse-radish peroxidase labelled antibodies were goat anti-rabbit (Sigma-Aldrich, A9169/1:10,000) or goat anti-rabbit (Santa Cruz sc-2004/1:6000). The bands were detected by a chemiluminescence WesternBright™ ECL Substrate kit (Advansta, Menlo Park, CA, USA). Bands were visualised and quantified by Tanon-5200 Chemiluminescent Imaging Analysis System (Tanon Science & Technology, Shanghai, China).

### 5.6. Statistical Analysis

One-way ANOVA was used to determine the effects of dietary ZEN on each variable within the same tissue. Duncan test was used for the post hoc analysis. All data are presented as the mean ± SD, and the significance level was set at *p* < 0.05. All analyses were conducted using IBM SPSS Statistics 19 (IBM Corporation, Armonk, New York, NY, USA).

## Figures and Tables

**Table 1:** Effects of dietary zearalenone (ZEN) on feed consumption, body weight gain, and reproductive performance of pregnant rats ^1^.

Parameters	Treatments			
	Control	5 ZEN	10 ZEN	20 ZEN
Initial BW	206.39 ± 3.22	205.90 ± 6.65	206.30 ± 3.86	205.47 ± 5.34
Final BW	318.70 ± 18.85 ^a^	303.62 ± 19.51 ^ab^	286.63 ± 12.40 ^bc^	274.12 ± 8.16 ^c^
ADI, g/day	24.79 ± 1.29 ^a^	24.02 ± 1.81 ^a^	22.57 ± 2.11 ^ab^	19.23 ± 1.24 ^b^
ADG, g/day	5.12 ± 1.03 ^a^	4.87 ± 0.68 ^ab^	4.62 ± 0.41 ^b^	3.85 ± 0.45 ^c^
Litter size	11.52 ± 1.89	11.58 ± 2.57	10.92 ± 2.07	10.20 ± 1.48
Viable newborns	11.47 ± 1.36 ^a^	11.41 ± 1.31 ^a^	10.08 ± 1.83 ^ab^	9.60 ± 1.14 ^b^
Viability	0.99 ± 0.03 ^a^	0.91 ± 0.26 ^a^	0.89 ± 0.27 ^ab^	0.86 ± 0.08 ^b^

^1^ Values are expressed as means ± SD, *n* = 12. ^a,b,c^ Values in a row without a common superscript letter are significantly different, *p* < 0.05. ZEN, zearalenone; Control, 0 ZEN diet; 5 ZEN, 5 mg/kg ZEN diet; 10 ZEN, 10 mg/kg ZEN diet; 20 ZEN, 20 mg/kg ZEN diet. BW, body weight; ADI, average daily intake; ADG, average daily gain.

**Table 2:** Effects on growth performance of F1 female rats ^1^.

Parameters	Treatments			
	Control	5 ZEN	10 ZEN	20 ZEN
Neonatal (d 0)	5.74 ± 0.63 ^a^	5.27 ± 0.74 ^a^	5.19 ± 0.62 ^a^	4.70 ± 0.82 ^b^
Weaned (d 21)	35.46 ± 2.14 ^a^	34.03 ± 1.39 ^a^	31.84 ± 2.00 ^ab^	26.75 ± 4.40 ^b^
Adult (d 63)	204.23 ± 5.75 ^a^	193.15 ± 12.97 ^a^	178.06 ± 4.63 ^bc^	165.57 ± 10.79 ^c^
ADI, g/day	18.91 ± 1.44 ^a^	18.88 ± 1.39 ^a^	18.10 ± 1.09 ^a^	17.80 ± 1.40 ^b^
ADG, g/day	4.38 ± 0.63 ^a^	4.07 ± 0.74 ^a^	3.19 ± 0.62 ^ab^	2.70 ± 0.82 ^b^

^1^ Values are expressed as means ± SD, *n* = 12. ^a,b,c^ Values in a row without a common superscript letter are significantly different, *p* < 0.05. ZEN, zearalenone; Control, 0 ZEN diet; 5 ZEN, 5 mg/kg ZEN diet; 10 ZEN, 10 mg/kg ZEN diet; 20 ZEN, 20 mg/kg ZEN diet. ADI, average daily intake; ADG, average daily gain.

**Table 3:** Concentrations of FSH, LH, and E_2_ in the serum of maternal and F1 female rats ^1^.

Parameters	Treatments			
	Control	5 ZEN	10 ZEN	20 ZEN
**Maternal**				
FSH, IU/L	7.17 ± 1.78 ^b^	8.48 ± 2.09 ^ab^	10.52 ± 1.92 ^ab^	12.25 ± 1.42 ^a^
LH, ng/mL	1.84 ± 0.34	2.08 ± 0.43	1.87 ± 0.45	1.85 ± 0.10
E_2_, ng/L	75.78 ± 1.28 ^a^	74.44 ± 2.11 ^a^	70.67 ± 1.07 ^b^	66.66 ± 1.08 ^c^
**Adult Female**				
FSH, IU/L	4.59 ± 0.43 ^b^	7.07 ± 2.41 ^ab^	6.72 ± 0.60 ^ab^	7.93 ± 1.75 ^a^
LH, ng/mL	1.31 ± 0.25 ^b^	1.53 ± 0.27 ^b^	2.37 ± 0.57 ^ab^	2.57 ± 0.49 ^a^
E_2_, ng/L	61.49 ± 3.39 ^a^	58.99 ± 2.73 ^ab^	55.97 ± 0.95 ^b^	51.70 ± 0.79 ^c^

^1^ Values are means ± SD, *n* = 5. ^a,b,c^ Means within a line with different superscripts are significantly different at *p* < 0.05. Abbreviations: FSH, follicle-stimulating hormone; LH, luteinizing hormone; E_2_, oestradiol; ZEN, zearalenone; Control, 0 ZEN diet; 5 ZEN, 5 mg/kg ZEN diet; 10 ZEN, 10 mg/kg ZEN diet; 20 ZEN, 20 mg/kg ZEN diet.

## Supplementary Materials

The following are available online at www.mdpi.com/2072-6651/9/1/21/s1, Table S1: Relative organ weights (%) of adult F1 female rats, Table S2: ZEN concentrations in feed and the number of treated rat per group, Table S3: Gene sequences used in the present study.
