# Supplementary material for: Gestational Zearalenone Exposure Causes Reproductive and Developmental Toxicity in Pregnant Rats and Female Offspring
Source: Toxins (Basel). 2017 Jan 5;9(1):21. doi: 10.3390/toxins9010021 (PMC5308253; doi:10.3390/toxins9010021)
Supplement: Supplementary file 1 [file toxins-09-00021-s001.pdf]

Supplementary Materials: Gestational Zearalenone Exposure Causes Reproductive and Developmental Toxicity in Pregnant Rats and Female Offspring

Xin Gao, Lvhui Sun, Niya Zhang, Chong Li, Jiacai Zhang, Zhuohui Xiao and Desheng Qi

Table S1. Relative organ weights (%) of adult F1 female rats [1].

Organs	Treatments			
	Control	5 ZEN	10 ZEN	20 ZEN
ovary	0.056 ± 0.013 [a]	0.059 ± 0.003 [a]	0.057 ± 0.012 [a]	0.053 ± 0.007 [a]
uterus	0.22 ± 0.01 [a]	0.21 ± 0.07 [a]	0.14 ± 0.04 [b]	0.14 ± 0.03 [b]
liver	4.21 ± 0.31 [a]	4.18 ± 0.36 [a]	4.01 ± 0.26 [a]	4.33 ± 0.29 [a]

[1] Values are expressed as means ± SD, $n = 12$. Values in a row without a common superscript letter are significantly different, $p < 0.05$. ZEN, zearalenone; Control, 0 ZEN diet; 5 ZEN, 5 mg/kg ZEN diet; 10 ZEN, 10 mg/kg ZEN diet; 20 ZEN, 20 mg/kg ZEN diet.

Table S2. ZEN concentrations in feed and the number of treated rat per group [1].

Treatments	Concentrations of ZEN in Diets, mg/kg		Number of Rats
	Supplementation	Analysed Concentration	
0 ZEN (control)	0	0.01 ± 0.00	16
5 ZEN	5	4.82 ± 0.36	16
10 ZEN	10	9.21 ± 1.11	16
20 ZEN	20	19.54 ± 2.65	16

[1] ZEN, zearalenone. Concentrations of ZEN in diet were determined with high-performance liquid chromatography method according to *AOAC* [1].

Table S3. Gene sequences used in the present study.

Gene Name	Primer Sequence	Amplification Length (bp)	GenBank Accession No.
Esr1	Forward, 5′ AAGGAGACTCGCTACTGTGC 3′<br>Reverse, 5′ TCGGCGGTCTTTTCGTAT 3′	243	NM_012689.1
GnRHr	Forward, 5′ GCTGGGCAGAAGAAGATGTAA 3′<br>Reverse, 5′ ACGCTGCTGGGTATAGAAATG 3′	134	NM_012767
3β-HSD	Forward, 5′ CAAATCCATACCCATACAGC 3′<br>Reverse, 5′ GCCACATTGCCTACATACAC 3′	235	L17138.1
ABCb1	Forward, 5′ AAAGCTATCACGGCCAACA 3′<br>Reverse, 5′ GATGACCAAGGAAGTCCCATAC 3′	96	NM_133401
ABCc1	Forward, 5′ GACCTTATTCAGTCCACCATCC 3′<br>Reverse, 5′ CCAGGACAATCACCCTTGTATAG 3′	106	NM_022281
ABCc5	Forward, 5′ GCCACCATCCATGCCTATAA 3′<br>Reverse, 5′ CATTGCACAGGTGAACAAGAAG 3′	102	NM_053924.1
GAPDH	Forward, 5′ GCAAGTTCAACGGCACAG 3′<br>Reverse, 5′ CGCCAGTAGACTCCACGAC 3′	141	NM_017008

References

1. Bennett, G.A.; Shotwell, O.L.; Kwolek, W.F. Liquid chromatographic determination of alpha-zearalenol and zearalenone in corn: collaborative study. *J. Assoc. Off. Anal. Chem.* **1985**, *68*, 958–961.
